# Influence of Underutilized Unripe Banana (*Cavendish*) Flour in the Formulation of Healthier *Chorizo*

**DOI:** 10.3390/foods10071486

**Published:** 2021-06-26

**Authors:** Diego Salazar, Mirari Arancibia, Karen Raza, María Elvira López-Caballero, Maria Pilar Montero

**Affiliations:** 1 Facultad de Ciencia e Ingeniería en Alimentos, Universidad Técnica de Ambato, Av. Los Chasquis y Rio Payamino, 180206 Ambato, Ecuador; marancibias@uta.edu.ec (M.A.); kraza1360@uta.edu.ec (K.R.); 2 Facultad de Veterinaria, Universidad Complutense de Madrid, 28040 Madrid, Spain; 3 Instituto de Ciencia y Tecnología de Alimentos y Nutrición (ICTAN-CSIC), Calle José Antonio Novais 10, 28040 Madrid, Spain; elvira.lopez@ictan.csic.es

**Keywords:** unripe banana flours, enriched fiber, reduced fat, *chorizo*, storage

## Abstract

The purpose of this work was to obtain chorizos by partially fat replacing with banana flour (whole or peeled). These *chorizos* were formulated with 3% pork fat and 24% whole banana flour (BC) or banana peel flour (BPC). A third formulation of chorizo with 15% pork fat and 12% wheat flour (WC) was also produced for comparison. Cooking loss was 12.5% for the WC, while for the BC and BPC *chorizos* it was 7.2% and 6.9%, respectively. All three products had similar water, protein, and ash contents, whereas carbohydrate and fiber contents were the main changes in composition. The color of the three different formulations did not change markedly, but an increase in yellowness and chromaticity was observed in the BC *chorizo*, as well as a slight decrease in lightness and in the whiteness index in the BPC one. Textural properties declined from day 0; from day 3 onwards, they remained constant in all *chorizos* and properties, except for BC lot in cohesiveness. Mesophilic aerobic bacteria, as well as mold and yeast counts, were predominantly high in the WC during chilled storage. Moreover, the sensory analysis indicated high acceptability of the formulated with wheat or whole banana flour, although those with banana peel flour scored slightly lower. This study shows that incorporating banana flours into the formulation successfully reduced the incorporation of pork back-fat, resulting in excellent quality sensorial characteristics due to the technological parameters and sensory acceptance.

## 1. Introduction

Nowadays, two of the most common health problems in society are cardiovascular disease and obesity, associated with high-fat intake. In this sense, consumers demand food industries develop, produce, and improve healthier and high-valuable products [1]. Despite this, fat-rich products, such as meat derivatives, continue to be highly popular among consumers of all generations. Thus, searching for formulations to develop healthier meat products continues to be a challenge. *Chorizo* is a meat product made of beef, pork, and pork back-fat, spiced with salt and additives, similar to some kind of sausages with coarsely ground meat, where composition may differ according to the regions. In general, these products are rich in fat (26 to 32%) [2,3], and neither fiber nor starch is included in the formulation. Traditionally, *chorizo* is stuffed in natural casings and may receive a maturation process before consumption [4,5] as a fermented cured product. Therefore, there are several meat products called *chorizo* (fresh, cooked, or cured), widely commercialized in numerous countries, especially Spain, Portugal, and Latin America.

Due to the high fat content, it would be advisable to reformulate *chorizo* with a reduced amount of fat to achieve a healthier product while maintaining the sensory properties, mainly in relation to taste and texture, which consumers are not willing to give up [6]. In this context, one of the main difficulties that researchers face when reformulating products is that fat plays a crucial function in stabilizing emulsions in meat products, and thus, decreasing cooking losses and consequently improving the sensorial characteristics [7,8]. Several researchers have conducted studies focused on reformulations to diminishing the high-fat percentage by partial substitution of fat with other ingredients that attempt to simulate similar sensory properties and even provide extra health benefits [9,10]. Among others, the ingredients most commonly utilized as fat substitutes in meat products and cooked sausages include proteins [6], hydrocolloids such as carrageenans, tragacanth [11], fibers, and flours [12,13,14], and even unsaturated fats or oils [15,16]. However, in most cases, technological and sensory limitations do not allow reaching standard product characteristics. The addition of dietary fiber (such as wheat fiber) in gel-emulsion-type meat products becomes more interesting when it is mixed by pre-emulsifying with some water, which favors the retention of water and lipid-binding properties [11,12,17]. However, the quantities of fat replacers added in gel-emulsion meat products are not very high (3%), [17], 4% [18], although higher amounts were reported using blends (*punica granatum and citrus spp. extracts* (10%)) [19], or banana flour (12%) [20] showing good characteristics and sensorial properties. On the other hand, the search for nutritional and healthy ingredients, which also represent an essential valorization of the resources and by-products of the agri-food industry, is a bet for the future in food research. Based on this, banana flour has been identified as an ingredient of great interest.

Banana growing has become the primary basis of the economy in some Latin American and Asian countries [16]. Banana exports in Latin America and the Caribbean stood at 15.1 million tons during 2019. Ecuador represents more than 40% of the region’s exports, and with Guatemala and Colombia (the second and fourth largest exporters, respectively), generates an important economic movement worldwide (FAO, 2019). However, fruit that is not suitable for export is used in agribusiness, for animal feed, or fresh fruit for national markets. In these countries, the amounts for rejected green bananas are estimated between 5% and 10% [21]. The most used flour in the world is from wheat, and Latin American countries account for approximately 99% (600,000 tons) [22]. Although green bananas are used for domestic consumption, they are still an underutilized product, and the search for diverse food applications, for example, flours, is a challenge in many countries around the world. In addition to considering the use of bananas to obtain flour for reasons of price appreciation and trade, it should be noted that both unripe banana flours (whole and with peel) have a high nutritional value (high fiber, mineral, and resistant starch content) [23,24]. The green banana peel constitutes 35% of the total fruit weight. However, the peel is also an underutilized product with potential benefits for human health. Moreover, the antioxidant activity in the peel is much higher than in the pulp [23]. In popular medicine, the banana peel has been used to treat several sicknesses and disorders such as abscess, intestinal disorders, inflammation, diabetes, anemia, or even snake bites [25]. The peel has a high amount of protein, fat, and fiber, which makes it particularly suitable for several applications in food products [26,27].

Gel-emulsion-type meat products have been elaborated with the addition of banana flour, such as Frankfurter-type sausages [20], or by the addition of gelatin-banana flour–water mixture (1:2:2) in Bologna-type sausages [28]. However, there are no reports about their use in *chorizo* formulations. Their techno-functional characteristics can be very different since in *chorizo*, the meat does not entirely lose its integrity and the emulsion produced with the fat present is not stable; the addition of flour, fiber, or other compounds could show a different behavior, producing a decrease in particle size that causes a loss of palatability [18]. The nutritional value of banana flour is an advantage that brings into consideration substituting the habitually used food technology wheat flour with this new product since gluten intolerance grows daily in the population [29].

This study aims to develop and evaluate a new type of *chorizos*. These products will be formulated to reduce fat and enriched fiber with the incorporation of unripe underutilized banana flours (whole or peel). *Chorizo* formulated with wheat flour, widely used in food technology, was also produced for comparison purposes. Therefore, in view of these nutritional considerations, it is expected to obtain new healthier meat products, some of them suitable for coeliacs, with a proper taste and desirable sensory properties.

## 2. Materials and Methods

### 2.1. Elaboration of Banana Flour

Green (unripe) bananas were acquired in a traditional market in Ambato (Ecuador). The bananas were chosen in base to uniformity and homogenous color. The fruits were exhaustively washed in water. For the development of the study, the whole bananas were cut into slices about 3 mm thick and 3.30 cm in diameter. To obtain the peel flour, fruits were skinned, and the peel was cut with a thickness and length of about 0.5 cm. All samples were evenly placed in platters in a convective dryer (Gander MTN, Saint Paul, MN, USA) at 60 °C for ~24 h (around 12% moisture). Once dry, the slices of each bath were crushed in an industrial mill (Inox Equip IE1, Riobamba, Ecuador) to obtain the flours. Both whole banana flour and banana peel flour were stored in bags, hermetically closed, and keep at room temperature until use.

### 2.2. Preparation of Chorizos

**Figure 1:**
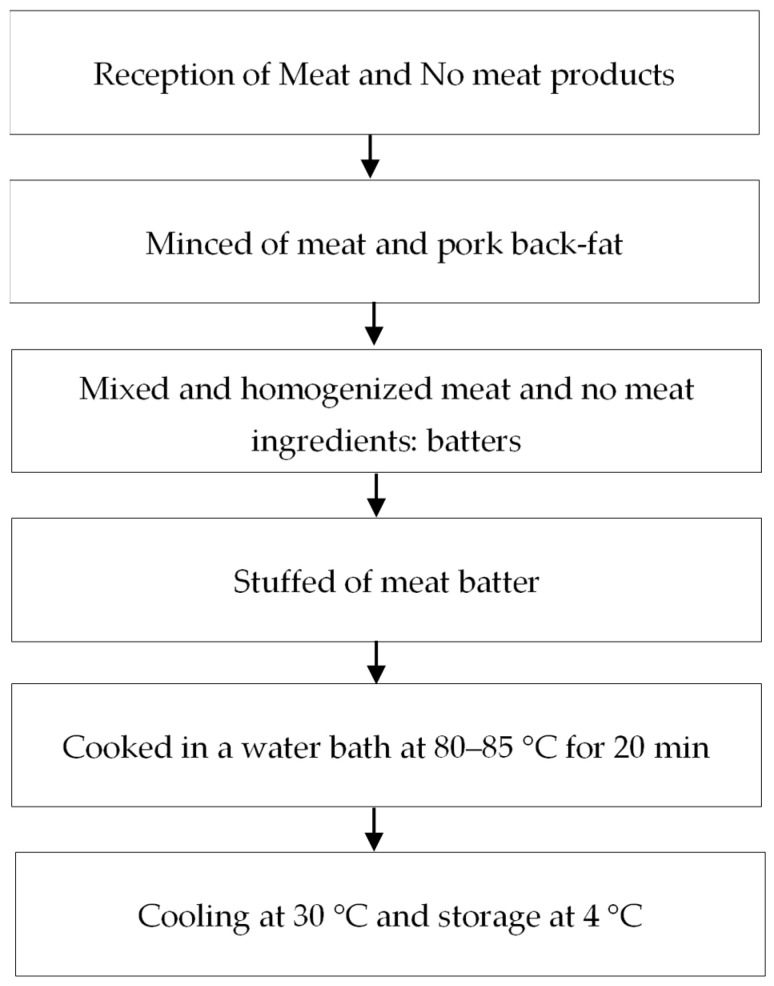
Chorizo procedure scheme.

The *chorizo* was made following a standard manufacturing procedure (Figure 1). The basis of the formulation was similar in the three formulas and consisted of meat protein and no meat products with the same proportion in all formulas; the exception was a variable fat and flour content, adding a different type of flour in each formula. Formulations are shown in Table 1. The compositions of flours were: wheat flour (moisture 14%, protein 12%, fat 2%, ash 0.65%, fiber 2.75%), whole banana flour (moisture 11.32%, protein 3.53%, fat 0.87%, ash 3.76%, fiber 3.51%), banana peel flour (moisture 9.64%, protein 4.24%, fat 3.51%, ash 6.35%, fiber 10.26%). The analysis was carried out according to official methods of AOAC (AOAC, 2000) [30].

The meat and fat were minced using a hole plate of 0.5 mm in diameter. Then, they were mixed together with 4.5% ice for 30 min in a mixer (Mainca CM.21, Barcelona, Spain); afterward, the other ingredients were added and homogenized for 2 min at chilled temperature (<5 °C) to obtain a thick homogeneous paste. Later, mixed meat batter was stuffed into a natural casing (Alico, Medellin, Colombia) of approximately 24 mm diameter and 50 mm length; after that, all samples were cooked in a water bath at 80–85 °C for 20 min until achieving 73 °C in the center of chorizo. Then, they were kept in a cold-water bath until 30 °C in the center of the piece. Finally, *chorizos* were packed in high-density polyethylene plastic bags and kept at 4 °C until analysis. Three experiments from each batch of *Chorizo* were carried out.

### 2.3. Cooking Loss

The cooking loss during heat treatment (1 kg per batch) was determined. The analysis was carried out in triplicate and the calculation was made using the formula: cooking loss (%) = ((chorizo weight before cooked − chorizo weight after cooked)/chorizo weight before cooked) × 100.

### 2.4. Proximal Analysis

Proximate composition (moisture, ash, protein, and fat) was evaluated following the official methods AOAC 19 927.05, AOAC 923.03, AOAC 2001.11, and AOAC 2033.06, respectively. Carbohydrate content was estimated by difference. Protein content was estimated by nitrogen determination using the factor of 6.25. The dietary fiber determination was made by enzymatic–gravimetric method (AOAC 985.29) (PRT-701.03-019, 2011). All assays were made in triplicate, and the results were expressed as a percentage.

### 2.5. Energy Value

Calorie content was calculated by 100 g, being the total sum of calories of the individual components. The component energy value is as follows: protein (×4 kcal/g), fat (×9 kcal/g), carbohydrate (×4 kcal/g), and fiber (×2 kcal/g) contents.

### 2.6. Acidity and pH

The pH of the batches was measured with a pH meter (HANNA HI 9126, Woonsocket, RI, USA). The acidity was analyzed by titration with NaOH 0.1N, and as an indicator, phenolphthalein was used, according to AOAC (2005). The data are expressed as a lactic acid percentage. All analyses were conducted in triplicate.

### 2.7. Microbiological Analysis

Ten grams of *chorizo* were aseptically placed into a sterile stomacher bag. They were then homogenized with 90 mL of buffered 0.1% peptone water (Difco, Le Pont de Claix, France) in a stomacher homogenizer (Model 400C, Seward, London, United Kingdom) for 1 min at room temperature. For each sample, appropriate serial decimal dilutions were prepared in peptone water. Mold and yeast were incubated at 25 °C and evaluated using Rose Bengal Agar (RBC) (Difco). Viable aerobic mesophilic microorganisms in PCA agar (Difco) were incubated at 30 °C for 72 h. *Enterobacteriaceae* on a double layer of Violet Red Bile Glucose Agar (VRBG) were incubated under the same conditions (Acumedia, Lansing, MI, USA), and *Staphylococcus aureus* on Baird Parker agar (Difco) with egg yolk tellurite supplement were incubated at 30 °C for 48 h. All analyses were performed in triplicate. The tests were conducted in the course of 5 days.

### 2.8. Texture

The texture profile analysis (TPA) was performed in a texturometer (CT3 Brookfield, Scarsdale, NY, USA). Samples were cut into cubes of 1.0 cm high, 1.5 cm longitude, and 1.5 cm wide. A double compression with 25% deformation (normal stress) with 5″ compressions waiting period was made; a head speed of 1 mm/s, and a 25 kg load cell was used. The parameters to be measured were hardness (N), cohesiveness (dimensionless), springiness (mm), and chewiness (Nmm). Hardness is considered as the maximum force of the first compression cycle. Cohesiveness corresponds to the quotient between the positive areas under the force curve of the second compression. Springiness is the height that the food sample recovers during the time elapsed between the ends of the first compression until the start of the second one. Chewiness is the product of hardness, cohesiveness, and elasticity.

### 2.9. Color

The color parameters L* (lightness), a* (red/green), and b* (yellow/blue) were measured with a Hunter Lab Colorimeter (mini Scan 4500L EZ, Hunter Associates Laboratory INC, Reston, VA, USA) calibrated with an illuminator D65 (natural light) and standard observer D10. The results were expressed as Hue and Chroma values.

Saturation C* was determined from the equation C* = √ (a*^2^ + b*^2^) [31]. Furthermore, the whiteness index was determined according the equation W = 100 – ((100 − L*)^2^ + (a*^2^ + b*^2^))^1/2^ [32]. The white tile standard was used for color calibration. The data were obtained from different sample areas; at least 15 measurements were conducted, and the average registered is the indicated value.

### 2.10. Sensory Analysis

A sensory acceptance test was performed using a 5-point hedonic scale (5—liked very much; 4—like moderately; 3—neither liked nor disliked; 2—disliked moderately; 1—disliked very much). A panel of 20 semi-trained judges who are part of the team of athletes from the Technical University of Ambato assessed sensory attributes such as odor, taste, texture, and overall acceptability. Two slices (2.4 cm in diameter and 0.3 cm thick) of grilled samples without casing were provided; to rinse the palate, salted crackers and a glass of water were supplied.

### 2.11. Experimental Analysis

The experimental design of a single completely randomized factor was applied. Statistical analysis was conducted with the GraphPad Prism 5.0 program (GraphPad Software, San Diego, CA, USA) for the one-way ANOVA analysis. The pairwise comparison was carried out using the Tukey test (α = 0.05), with a significance level of *p* ≤ 0.05. The only design factor was flour with three levels: without banana flour, whole banana, and peel.

## 3. Results and Discussion

### 3.1. Compositional Analysis

The cooking loss of *chorizo* was 12.5 ± 1.5, 7.2 ± 1.3, and 6.9 ± 0.8% for WC, BC, and BPC, respectively. These values are mainly attributed to fat loss since fat was visible in the cooking water, and the values found in the final composition indicated this as well (Table 2). The results showed that the emulsion formed is very unstable, probably due to the ground meat and scarce homogenization characteristics of the product. A loss of fat is expected in this type of product during cooking, hence the incorporation of flour as a binder. BC showed more fat retention than BPC, while WC chorizo registered the highest cooking loss value, probably attributable to the high amount of fat that was used in the formulation of this chorizo. In previous work, the cooking loss was around 5.35–4.41% in gel-emulsion-type meat products added with banana flours in similar concentrations to those used in the present work [20].

Considering that the difference in fiber between the WC and BC *chorizos* is less than 1% and that the most significant differences are attributed to the non-fiber carbohydrate content (4.19% and 10.72% for WC and BC, respectively), the ability to trap fat must be attributed to the latter. With the exception of the work of Salazar et al. [20] in Frankfurter-type sausages, no information has been found in the literature on a meat product, with or without the incorporation of banana flour, so the present work results have been compared with other heat-treated meat products. In general, fibers and starch do not have the same properties; but as more quantity is incorporated, less cooking loss is expected [33]. Alves et al. [28] observed that with a greater amount of banana flour (4%) incorporated in a gel as a fat substitute, the cooking loss decreased in bologna-type sausages, and the percentage of fat exudation from the emulsion was much lower than in the WC sausages. According to Almeida et al. [24], the content of resistant starch in banana flours is much higher than in other varieties; particularly, the *Prata* variety of *Musa* sp. has above 37%, although in other varieties of Musa sp. ranges from 27% to 67.5%, while in wheat starch, it can be around 13.6% [34]. As well, differences in resistant starch percentages may also be found depending on the extraction method and crop variety and conditions. Those authors also indicate that heat treatment in water favors the increase of resistant starch in banana flour, reinforcing not only amylose–amylose and amylose–amylopectin interaction, but also amylose–lipid interactions, which some authors propose as type five [35], thus avoiding fat being exuded and, at the same time, promoting a better integration into the product. No drip was observed during chilled storage.

In the present work, the WC *chorizo* lost a large amount of fat in the cooking water despite the wheat flour content of the product, which favors its retention. This behavior was attributed to a greater fat in the formula, thus its release was facilitated. Achieving a formula in which no ingredients are lost during cooking is an interesting challenge at an industrial level. Choi et al. [12] showed that Frankfurters containing 2% rice bran fiber showed less cooking loss (*p* < 0.05) than products without added fiber. Meat products seem to have enhanced water-holding ability and emulsion stability when they contain fiber, which leads to a greater yield after cooking. In the present work, many experiments were conducted previously in order to define the proper ingredient concentrations. Thus, it was observed that wheat fiber does not allow addition in larger quantities without detriment to the quality of the *chorizo*; however, banana flour allows addition in higher concentrations. Therefore, three very different formulations were designed in order to assess their characteristics as meat products *chorizos*.

The *chorizo* containing banana peel flour (BPC) showed a greater moisture content (*p* < 0.05), which may be because of its greater fiber content (Table 2). Among the benefits of fiber is its ability to retain water [36,37]. In other studies in which meat products do not contain fiber, its addition makes water retention noticeable [38]. In this study, all *chorizos* contained more than 4% fiber; thus, there was no difference in water retention between the WC and BC *chorizos*; however, the higher fiber content of the BPC batch (3.7%) leads to a 4.9% increase in water content. According to Henning et al. [39], a humidity range between 58.1% and 68.1% is appropriate to obtain suitable properties to reduce fat in sausage formulations, being that fat replaced with different pineapple dietary fibers. The *chorizo* of this work was also included in this range. The fat content reduction in *chorizos* was 41.4% and 35% for the BC and BPC *chorizos*, respectively (Table 2). Based on the proposed formulation, a 50% reduction in fat content was expected, although due to the loss of compounds (mainly fat) in the WC *chorizo* during cooking, the reduction was lower. Moreover, it was observed that the BC and BPC samples barely lost fat during the cooking process, considering the fat values found in the final products (Table 2).

Regarding ash content, the *chorizos* were very similar and did not differ by more than 0.38% (*p* < 0.05). These values are in the same range as those obtained by Alves et al. [28], with values between 3.07% and 3.91% for low-fat sausages in which a percentage of fat was changed by a gel prepared with pork skin, water, and green banana flour in a ratio of 1:2:2 (20, 40, 60, 80, 100%). Choi et al. [12] indicated that the ash content increases according to fiber concentration within the meat matrix. In this regard, the highest ash content could be due to the presence of minerals such as sodium, potassium, calcium, and magnesium from flours [40,41,42].

The protein content of the WC *chorizo* was high (*p* < 0.05), while the batch with banana flours showed a slightly lower content (~1 and 2% less for BC and BPC, respectively). These results could be because of the greater protein content in wheat flour compared with banana flour (although the amount of wheat flour added was half the amount of banana flour in the formulas).

Carbohydrate content is markedly increased with the diminishing of fat, especially in the *chorizo* containing whole banana flour (whose content is about double that in the WC), with a starch content of ~10.72% for the BC *chorizo* and 2.6% for the BPC one, being the rest of carbohydrates fiber. As mentioned before, green banana flour starch can have resistant starch [24], in a considerably higher amount compared with wheat flour [34], which could enrich the nutritional properties of the BC and BPC products. Finally, the dietary fiber content showed a significant difference in all treatments. Fiber content increased according to the fiber concentration in each flour used for product development [43]. According to the nutrition claim related to fiber, foods must contain ≥6 g of fiber per 100 g to be labeled as “high fiber,” while foods containing ≥3 g of fiber per 100 of food should be labeled as “ a source of fiber” (EU Regulation 1924/2006) [44]. Many studies whose strategy is based on fat substitution through the addition of fiber do not usually raise the fiber content above 3% to avoid adverse effects on sensory quality [2,36,45]. In the present work, *chorizos* with a significant concentration of fiber in the formulation were successfully produced. Thus, the WC *chorizo* could be labeled as a “source of fiber” as well as the BC *chorizo*, while the BPC one could be labeled as “high in fiber content”. Other authors have also incorporated high amounts of fiber into various meat products, such as Chinese sausages, with ~3.5 oat fiber or wheat ~4% [46].

Regarding the caloric content after cooking, both *chorizos* with banana flours registered lower values than the WC (~13% and ~25% in the BC and BPC *chorizos*, respectively) (*p* < 0.05). Similar energy values to the ones of the BC *chorizo* were found in fat-reduced formulations of Frankfurters (by using a blend of oils from olive, linseed, and fish stabilized in two kinds of structures, an emulsion (oil in water), or a gel made with konjac, whose caloric content of 235 Kcal/100 g was reduced by up to ~165 Kcal/100 g [47]. Similarly, a high caloric content reduction was achieved (from 309.75 Kcal/100 g up to 139.30 Kcal/100 g) by replacing fat with fiber from makgeolli lees in Frankfurters [38].

The World Health Organization [48] recommended that, for a balanced diet, proteins should account for 10–15% of the total diet energy, carbohydrates around 55–75%, while the fat-percentage intake should constitute 15–30%, of which no more than 10% should come from saturated fats. Of course, these quantities are referred to as daily food intake, but meat products still constitute an important consumption source in some populations nowadays. The proportion of the energetic value of carbohydrates in relation to fat in the lower-fat *chorizos* is more balanced than in the WC *chorizo*, although the contribution of carbohydrates and fiber, in terms of energy, is much lower than would be desirable in order to achieve a better balance of the components. In commercial meat products, the percentage of fat is frequently quite high (40–50%), and the presence of carbohydrates is negligible, which is attributed to the presence of onion and rice in the same meat products, being the energy ratio is unbalanced [49].

Regarding pH, there are hardly any variations between samples (Table 2). Other authors have found lower values (6.30 to 6.63) in reduced-fat sausages with pea flour [37]. Several factors could affect the pH values in meat products; for example, the meat product could suffer a pH increment throughout the cooking process caused by histidine (basic amino acid) exposure [38], as well, reducing the fat content (<20%) in sausages generates lower pH values [38]. In the present work, the pH of the flours could modify the pH of the formulas to a certain extent, so wheat flour used in the WC sample has a pH of 6.3, while whole banana flours range between 4.30 and 5.65 [23].

On the other hand, acidity showed similar values among samples (*p* < 0.05). The acidity values measured at the end of cooking may be related to this process, given that pH (low acidity) could have increased, probably due to free-fat liberation and degradation of the cellular buffer [50,51].

### 3.2. Microbiological Analysis

**Figure 2:**
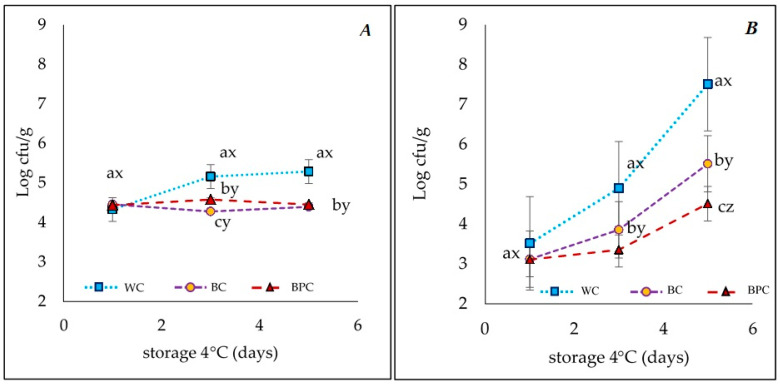
(**A**)Total aerobic mesophile bacteria [cfu/g] and (**B**) mold and yeast [cfu/g] during chilled storage of *chorizo*: WC (*chorizo* with wheat flour), BC (*chorizo* with banana flour), BPC (*chorizo* with banana peel flour). Values are the mean ± standard deviation. One-way ANOVA: different letters (a, b, c) in the same line show significant differences among days for each batch (*p* < 0.05). Different letters (x, y, z,.) in the same days show significant differences among batches for each day (*p* < 0.05).

Results of mesophilic aerobic bacteria during chilled storage are shown in Figure 2A. Initially, the three samples registered about 4 log cfu/g. Ranucci et al. (2019) reported 3.05 log cfu/g (total viable counts at 30 °C) in sausages made from pork meat, almond (*Prunus dulcis Mill*.), emmer wheat, and other dried fruits behind 1 day at 4 °C. Moreover, 3.86 cfu/g (aerobic mesophilic counts) values have been found in a beef Frankfurter-type sausage freshly made [52]. Counts increased in the WC *chorizo*, exceeding 5 log cfu/g in 6 days, while in the lower-fat *chorizo* remained more or less constant (*p* < 0.05). In chicken sausages with low fat content, Andrés et al. [53] observed lower counts (total mesophilic and psychrotrophic aerobic and lactic acid bacteria) in 5% added-fat sausages compared with 0% or 2% added-fat sausages, which was explained because products with a higher fat composition contained lower moisture. Despite the similar initial moisture values between WC and BC, and slightly higher in BPC (Table 2), perhaps the greater fiber content of lower-fat *chorizos* (BC and BPC) retained more water during storage, therefore being less available for microorganisms (Figure 2). Hayes et al. [54], reported values under 5 log CFU/g in pork luncheon roll samples containing tomato pulp at 14 days of chilled storage. However, Sharma et al. [55] documented a range of 3.21 to 6.91 log CFU/g during 15-day storage in fresh chicken sausages. *Enterobacteria* and *Staphylococcus aureus* were not detected in the three types of *chorizo* during the chilled storage.

Due to the presence of flours in the formulations, a mold and yeast count was performed on the *chorizo*. The initial values were equal in all samples (*p* < 0.05) (Figure 2B). During refrigerated storage, a progressive growth (*p* < 0.05) was observed in all treatments, although values were noticeably lower in both low-fat *chorizos* (*p* < 0.05). According to Sachindra et al. [56], the increase of molds and yeasts in this kind of meat product depends on the starch content of the specific flour since it is used as a substrate. In this sense, the highest growth corresponded to the WC, which contained wheat flour, being the percentage of starch ~49.24%, while for the *chorizos* with banana flour (whole or peel), starch contents were 67.85% and 25.77%, respectively. The low storage temperature (4 °C), together with the amount of starch available, generates a dominant effect of molds and yeasts in the microflora [57].

### 3.3. Textural Measurements

**Figure 3:**
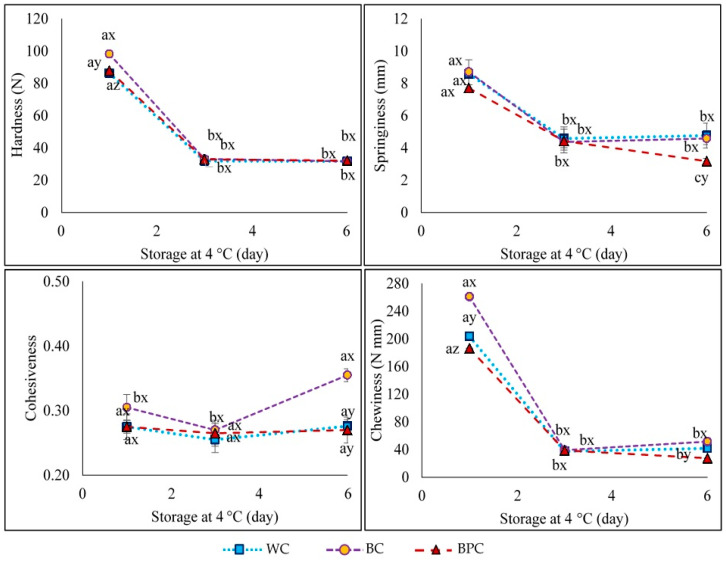
Textural properties of WC (*chorizo* with wheat flour), BC (*chorizo* with banana flour), BPC (*chorizo* with banana peel flour). Values are the mean ± standard deviation. Two-way ANOVA: different letters (a, b, c) show significant differences for each batch at different times (*p* < 0.05). Different letters (x, y, z) show significant differences among batches at same time (*p* < 0.05).

The textural profile analyses (TPA) of *chorizo* are shown in Figure 3. Regarding hardness, all samples were in the same range at day 0 despite formulation differences. This behavior could be extended to the rest of the texture parameters evaluated. Replacing fat with high-fiber or resistant starch content flours favors the maintenance of texture parameters. In this sense, Alves et al. [28] noted that high-fiber banana peel flour favors water retention, generating greater product hardness. Likewise, the presence of resistant starch in banana flour may favor water retention and improve texture [58]. Atashkar et al. [11], in sausages made of red meat and adding hydrocolloids as fat replacers (as tragacanth, κ-carrageenan, and konjac), described that the hardness is related to their fat and fiber content. These authors observed that the proteins of the product, together with fiber, produce a more rigid and firmer gel, leading to a drop in elasticity and a gain in the hardness and energy required for chewing. In the present work, the WC *chorizo* is a product that contains wheat flour, high in protein, starch, and fiber, which provides consistency and strength to the product. It has been challenging to achieve the same rheological property values for lower-fat *chorizos* considering such a different composition from the one of the WC. According to Varga-Visi et al. [33], the amount of fat reduced is balanced with the incorporation of flour, keeping approximately constant the amount of protein and water. However, this behavior is different from that reported by Choi et al. [36], who observed that the hardness, chewiness, and gumminess of tteokgalbi (a kind of Korean patty) increased with rising of rice bran fiber. However, in the mentioned work, some other constituents were modified in the formulation (i.e., higher water and protein contents in the low-fat-tteokgalbi compared with the high-fat one).

Concerning storage (4 °C), there was a significant drop in hardness, springiness, and chewiness in all samples, while no changes were found in the cohesiveness of the WC and BPC *chorizos* (Figure 3). These results show that the *chorizos* produced with green banana flours (BC and BPC) present a similar behavior to that of the WC lot. Moreover, the most frequent commercialization day for this type of product is around day 3, so the texture parameters already maintain much more stable values on this date, without the marked decrease in hardness observed from day 0. Yang et al. [59] reported that hardness and chewiness in oatmeal added pork sausages gradually decreased with the increasing addition of hydrated oatmeal, with a similar profile to those obtained in the present study.

### 3.4. Color

**Figure 4:**
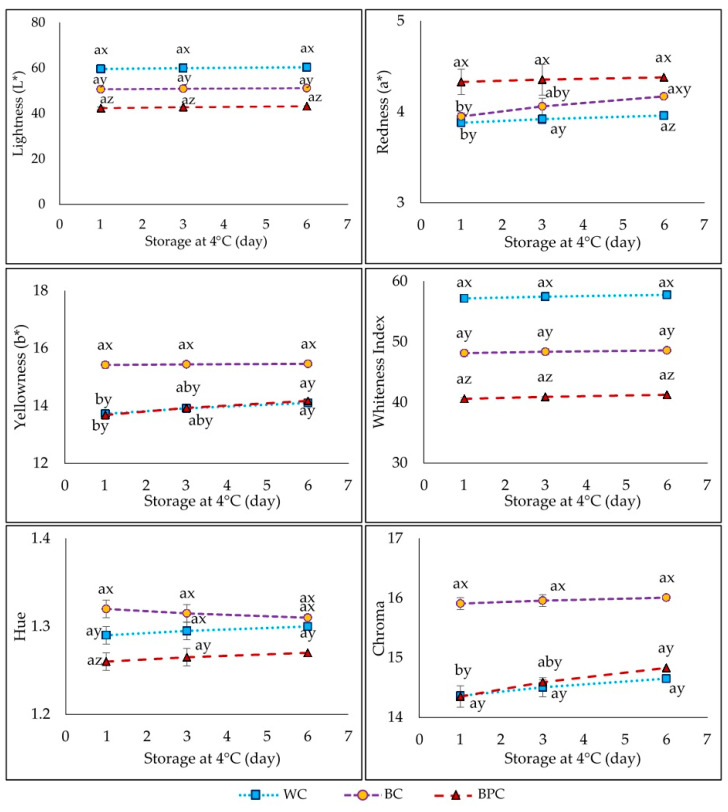
Influence of banana flours on the *chorizo* color parameters: WC *chorizo* with wheat flour), BC (*chorizo* with banana flour), BPC (*chorizo* with banana peel flour). Values are the mean ± standard deviation Two-way ANOVA: different letters (a, b, c) show significant differences for the same batch at different times (*p* < 0.05). Different letters (x, y, z) show significant differences among batches at same time (*p* < 0.05).

Banana flour moderately influences the color of lower-fat *chorizos* (Figure 4). Lightness diminished with the addition of whole banana flour and to a greater extent with banana peel flour (*p* < 0.05). On the first evaluation day, the highest redness (a*) values were those of the *chorizo* with banana peel flour, while a* maintained similar values (*p* < 0.05) in the other two formulas. Therefore, pulp presence in the whole banana flour minimizes the redness produced by the banana peel. This behavior was maintained throughout storage, and higher red values are probably due to the presence of salt. Conversely, on the first day, the yellowness (b*) values were different and higher in the whole banana flour sample, indicating that it is the pulp that most intensely manifests this coloration; a similar tendency was observed on the final day of storage. The lower lightness and the redness tendency because while drying, the banana peel suffered a browning phenomenon and the appearance of dark stains caused by the rupture of the peel chlorophyll during flour production [23], although the darkening of the samples could also be due to increased browning from Maillard reactions during the heat treatment. The whiteness index showed the same tendency as the luminosity (*p* < 0.05). Chromaticity and Hue presented values in a very similar range for all samples, although there was a slightly higher value of Croma in the BC *chorizo*, while in the hue angle, the value was slightly lower for the BPC *chorizo* (*p* < 0.05), albeit this value increased at the end of storage. Regarding the color properties of sausages, multiple results have been obtained by various authors [60], and they are related to the type of traditional product and the ingredients used as fat replacers.

### 3.5. Sensory Analysis

**Figure 5:**
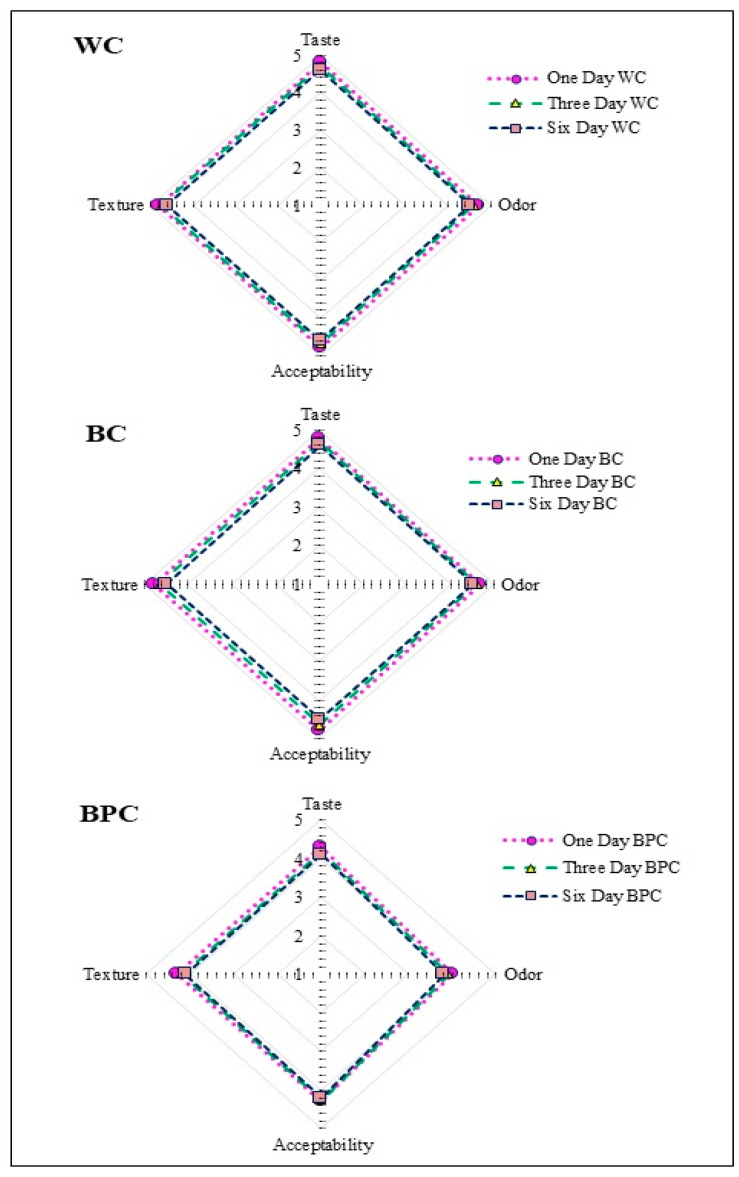
Sensorial evaluation of *chorizo* during first and sixth day. WC (*chorizo* with wheat flour), BC (*chorizo* with banana flour), BPC (*chorizo* with banana peel flour).

The results of the sensorial properties of the *chorizos* are shown in Figure 5. It is worth noting that the BC lot showed the same values on acceptability, odor, taste, and texture as the WC *chorizo* (*p* < 0.05); According to Yang et al. [59], the mixture of fiber and starch in banana flour and other minority compounds used in the *chorizo* contributed to enhancing sensory attributes. The difference of 5.5% of fat between WC and BC is not noticeable by the intrinsic characteristics of the whole banana flour, resulting in similar and palatable sensory properties. From a techno-functional point of view, this fact is also of great interest because it is possible adding more flour to partially replace fat. Choi et al. [36] found similar results when adding carrageenan and oatmeal flour as fat substitutes in sausages, which improved the texture, juiciness, and general acceptability of a reduced-fat sausage. On the other hand, the BPC sample showed values that were slightly lower in sensorial characteristics; the judges rated it above 3.5 for odor and taste, while in texture and acceptability, it was rated above 4. The evaluation on the last day of storage showed similar sensory attributes for the three *chorizos* as on the first day; only the odor of the BPC batch showed slightly lower values. On the last day, judges also rated sensorial attributes above 3.5, which means that they moderately liked the *chorizos*. The panelists did not describe any strange flavor; the *chorizo* containing the whole banana flour was slightly less tasty or different from what is usually consumed. Yang et al. [59] suggested that an excellent pork sausage with low fat content could be made substituting part of meat pork with hydrated oatmeal (≤25%). Incorporating dietary fiber (2%) into low-fat meat products helps maintain their quality characteristics [38]. Li et al. [35] suggest that whole banana flours, with pulp and peel, turn out to be excellent fat substitutes in hamburgers due to the increase in acceptability and to the improvement of the physical characteristics of the product. Thus, because of the results obtained (Figure 5), whole banana flour (pulp and peel) could be a very convenient fat substitute.

## 4. Conclusions

The inclusion of non-traditional carbohydrate sources as meat extenders is a viable alternative for meat products. The adding of wheat or banana flour (WC or BC, respectively) used in the formulation of *chorizo* could be a great strategy to improve the health and nutritional characteristics of *chorizos* with similar physico-chemical, textural and sensory properties. Cooking loss is diminished (~50%) in *chorizos* formulated with whole banana flour (BC) compared with the wheat *chorizo* (WC), which is of economic importance. In addition, BC showed a lower microbial load during chilled storage. Moreover, the presence of wheat flour may have limitations for consumers with allergenicity problems.

Regarding the *chorizo* in which banana peel flour was added, the sensorial properties were scarcely lower than in WC and BC; thus, it could also be a candidate for consumption. From a nutritional point of view, the fat reduction of >5.5% with the incorporation of both types of banana flour is important. Taking into account the amount of fiber incorporated in the respective formulations, only the BPC allows being labeled as a “high fiber” food, while WC and BC *chorizo* can be considered as a “source of fiber”. In *chorizos* with banana flours, the added energetic reduction in terms of Kcal/100 g has also been noticeable. In general, the health properties of both lower-fat products have been improved.

## Figures and Tables

**Table 1:** Chorizo formulations with different flours.

Ingredients	Treatments		
	WC (g/100 g)	BC (g/100 g)	BPC (g/100 g)
Beef	31	31	31
Pork	27	27	27
Frosty ice	9	9	9
Seasonings *	4.4	4.4	4.4
Sodium chloride	1.6	1.6	1.6
Pork back-fat	15	3	3
Wheat flour	12	-	-
Whole banana flour	-	24	-
Banana peel flour	-	-	24

* (180 mg/kg sodium nitrite, 0.05% polyphosphates, and 0.05% ascorbic acid powder, garlic powder, onion powder, pepper, nutmeg, cinnamon, and sugar).

**Table 2:** Proximate composition, caloric content (Kcal/100 g), pH, and acidity (as % of lactic acid), of chorizo: WC (chorizo with wheat flour), BC (Chorizo with banana flour), BPC (chorizo with banana peel flour).

Properties	WC	BC	BPC
Moisture (%)	62.8 ± 0.84 ^a^	62.3 ± 0.58 ^a^	67.7 ± 0.86 ^b^
Ash (%)	3.20 ± 0.05 ^a^	2.96 ± 0.05 ^b^	2.96 ± 0.05 ^b^
Protein (%)	12.05 ± 0.27 ^a^	11.06 ± 0.65 ^b^	10.16 ± 0.01 ^c^
Fat (%)	13.44 ± 0.57 ^a^	7.88 ± 0.65 ^b^	8.71 ± 0.46 ^c^
Total Carbohydrates (%)	8.51 ± 0.75 ^a^	15.8 ± 0.50 ^b^	10.09 ± 0.23 ^c^
Fiber (%)	4.32 ± 0.28 ^a^	5.08 ± 0.46 ^a^	7.49 ± 0.13 ^b^
Calories (Kcal/100 g)	194.54 ± 0.73 ^a^	168.37 ± 2.36 ^b^	144.24 ± 1.57 ^c^
Fat Calories (Kcal/100 g)	62.19 ± 0.49 ^a^	42.14 ± 0.63 ^c^	54.26 ± 0.85 ^b^
CH and F Calories (Kcal/100 g)	13.02 ± 0.59 ^c^	31.55 ± 0.34 ^a^	15.57 ± 0.29 ^b^
Protein Calories (Kcal/100 g)	24.80 ± 0.69 ^a^	26.31 ± 0.23 ^a^	28.17 ± 1.02 ^a^
pH	6.93 ± 0.05 ^a^	6.84 ± 0.04 ^b^	6.88 ± 0.03 ^a,b^
Acidity (%)	0.09 ± 0.05 ^a^	0.08 ± 0.01 ^a^	0.07 ± 0.01 ^a^

Values are the mean ± standard deviation. One-way ANOVA: different letters (a,b,…) in the same column show significant differences among batches (*p* < 0.05). F: fiber, CH: carbohydrate.
